# A Comprehensive Tutorial on *In Vitro* Characterization of New Photosensitizers for Photodynamic Antitumor Therapy and Photodynamic Inactivation of Microorganisms

**DOI:** 10.1155/2013/840417

**Published:** 2013-05-16

**Authors:** Tobias Kiesslich, Anita Gollmer, Tim Maisch, Mark Berneburg, Kristjan Plaetzer

**Affiliations:** ^1^Department of Internal Medicine I, Paracelsus Medical University/Salzburger Landeskliniken (SALK), Muellner Hauptstrasse 48, 5020 Salzburg, Austria; ^2^Institute of Physiology and Pathophysiology, Paracelsus Medical University, Strubergasse 21, 5020 Salzburg, Austria; ^3^Department of Dermatology, University Hospital Regensburg, Franz-Josef-Strauss-Allee 11, 93053 Regensburg, Germany; ^4^Department of Dermatology, Eberhard Karls University, Liebermeisterstraße 25, 72076 Tuebingen, Germany; ^5^Laboratory of Photodynamic Inactivation of Microorganisms, Department of Materials Science and Physics, University of Salzburg, Hellbrunnerstraße 34, 5020 Salzburg, Austria

## Abstract

*In vitro* research performed on eukaryotic or prokaryotic cell cultures usually represents the initial step for characterization of a novel photosensitizer (PS) intended for application in photodynamic therapy (PDT) of cancer or photodynamic inactivation (PDI) of microorganisms. Although many experimental steps of PS testing make use of the wide spectrum of methods readily employed in cell biology, special aspects of working with photoactive substances, such as the autofluorescence of the PS molecule or the requirement of light protection, need to be considered when performing *in vitro* experiments in PDT/PDI. This tutorial represents a comprehensive collection of operative instructions, by which, based on photochemical and photophysical properties of a PS, its uptake into cells, the intracellular localization and photodynamic action in both tumor cells and microorganisms novel photoactive molecules may be characterized for their suitability for PDT/PDI. Furthermore, it shall stimulate the efforts to expand the convincing benefits of photodynamic therapy and photodynamic inactivation within both established and new fields of applications and motivate scientists of all disciplines to get involved in photodynamic research.

## 1. Introduction

Photodynamic procedures combine three per se harmless components, namely, a light-sensitive molecule (the photosensitizer, PS), non-UV light corresponding to an absorption peak of the PS, and molecular oxygen to remove harmful pathogens, cells, or tissue(s). Within the last decades, the most prominent application of this approach, photodynamic therapy (PDT), has become a valuable alternative to classical treatment of localized malignant diseases since clinical studies prove its effectiveness, particularly in early stage tumors. The success of PDT is based on three mechanisms by which, either alone or in combination, the light-induced and PS-catalyzed overproduction of reactive oxygen species (ROS) destroys tumors: direct tumor cell death, damage set to the vasculature, and induction of a local inflammation with a subsequent immune response. For some indications (e.g., skin precancers and cancers) PDT represents a valuable therapeutic option also due to the excellent cosmetic outcome. Based on the design of new PS (with near infrared absorption), PS formulations, or PS/nanoparticle conjugates as well as technical improvements, still new applications of PDT are regularly identified [1]. 

Motivated by the achievements of recent PDT research and justified by the severe health threat that antimicrobial resistance poses to humans' health photodynamic inactivation of microorganisms (PDI) has been (re)introduced as revolutionary approach to kill bacteria, viruses, yeasts, and parasites. The lack of new antibiotics classes combined with the propagation of multidrug resistant bacteria/fungi and the economic and regulatory challenges thereof have boosted the research on PDI [2–4]. As the properties of ideal PS for PDT and PDI differ, numerous new substances especially synthesized for this promising approach are being developed in the photodynamic research community and expand the field of applications (e.g., to water-borne diseases) [5, 6].

Photodynamic therapy and PDI require, as multidisciplinary approaches, the tight cooperation of chemists (e.g., for synthesis of new PS), biologists (for testing new substances), physicists (e.g., for light dosimetry), and clinicians (for the transfer from the lab bench to the clinical application). The development of novel PDT/PDI applications in biomedicine and biotechnology is mainly driven by chemists and biologists. The former design new PS with promising properties, sometimes without the claim to intend the new substance for a specific target, and the latter test these new dyes *in vitro* on eukaryotic or prokaryotic cell cultures in order to estimate the possible field of application of a PS. Although many experimental steps of PS testing make use of the wide spectrum of methods readily employed in cell biology, some special aspects (e.g., fluorescence of the PS molecule or the requirement of subdued light conditions) have need of being considered when doing *in vitro* experiments in PDT/PDI. Up to date, the scientific photodynamic community did neither suggest a standard strategy for PS testing, nor does a tutorial on PS testing exist.

The aim of this paper is therefore to provide a comprehensive overview of experimental strategies and methods—without extensive referencing in order to maintain readability—by which novel photoactive drugs can be tested *in vitro* for their employment in photodynamic procedures. It shall represent a suggestion of operative instructions by which novel photoactive molecules may be identified as suitable for PDT and PDI, based on photochemical and photophysical properties of a PS, its uptake into cells, the intracellular localization, and photodynamic action in both tumor cells and microorganisms. This tutorial shall stimulate the efforts to expand the convincing benefits of photodynamic procedures within both established and new fields of applications and motivate (young) scientists to contribute to the photodynamic research. 

## 2. Characterization of the Photochemical and Photophysical Properties

### 2.1. Recording of Absorption/Fluorescence Spectra in Various Solvents/Cells

The absorption/excitation wavelength(s) of a given PS represents a key selection criterion for its application in PDT or PDI. As a common agreement, UV activation of photoactive drugs is inadmissible to exclude damage set to the cells' genetic information. Also, light wavelengths which are absorbed by the major tissue or cell chromophores should be avoided for excitation of the PS. For PDT on solid tumors, the effective penetration depth of the excitation light highly depends on the interference with the absorption spectra of the major tissue chromophores, namely (oxy-/deoxy-) hemoglobin and melanin, as well as water. The absorption spectra of these molecules define the optical window for PDT in tissue which covers the wavelength range of 600–850 nm [7]. The upper limit of this window is set by the minimal quantum energy, which is required for an efficient production of singlet oxygen, considering thermal loss of energy combined with the shifts of the electrons during the photophysical processes [8]. Due to a different composition of chromophores in microorganisms, the lower wavelength limit of the optical window defined for PDT applications does not necessarily apply for photosensitizers employed in PDI.

As the photophysical processes in a fluorescing molecule are dependent on the solvent, excitation and emission spectra of a PS should be read in aqueous solutions (buffers) or biocompatible solvents such as dimethyl sulphoxide (DMSO) or ethanol. In order to increase the water solubility of rather lipophilic PS, fetal calf/bovine serum (FC/BS) or other solubility enhancers such as polyvinylpyrrolidone [9, 10] may be added for recording of the spectra. In very rare cases (e.g., for photosensitizers showing absorption peaks with a narrow spectral half-width in combination with laser light illumination) the recording of spectra of the photosensitizer inside the cells might be necessary to assure a wavelength overlap of the light used for excitation with the absorption of the dye. Here, cells are incubated with the photosensitizer for a sufficient period of time, detached from the surface of the cell culture receptacle (e.g., by trypsinization) and washed with buffer to remove PS not internalized into cells. Fluorescence spectrometers allowing for stirring the solution of cells inside the cuvettes might be necessary and the spectra have to be corrected by samples with cells and without PS [11, 12]. 

Additionally, the experimenter has to take into account that not only the primary PS itself but also secondary molecules with different absorption resulting from photomodification of the primary PS may contribute to the overall photodynamic efficiency [11, 13].

The preparation of stock solutions of the photosensitizing agents might prove useful, as they might, depending on the chemical stability of the photosensitizer and the storage conditions, allow for a good experiment-to-experiment reproducibility of the PS concentration in PDT experiments. If stock solutions are prepared, the final concentration of solvents other than physiological buffers in the *in vitro* application should be as low as possible (for DMSO and ethanol <1% v/v), and the possible cytotoxic effect of the solvent should be tested by means of a viability assay (see the following chapter) under conditions identical to those used for incubation of the PS with the target cells. Storage of the PS should be performed according to the manufacturer's recommendations or may require individual optimization. The stock solutions of some PS may be kept frozen (exception: e.g., liposomal formulations). Independent of the storage conditions (as solid powder or in solution) the control measurements of the PS spectral properties on a regular basis are highly recommended to rule out PS decomposition.

### 2.2. Monitoring of Singlet Oxygen Production

**Figure 1:**
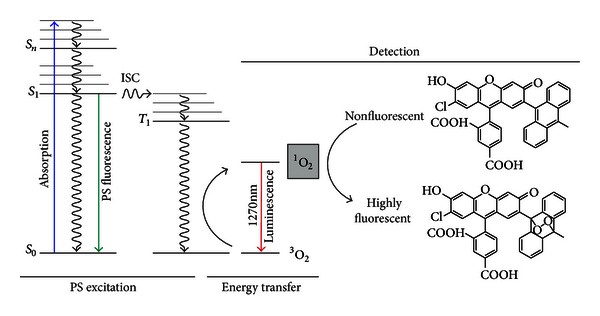
Jablonski diagram for the photosensitized production of ^1^O_2_ (left side) and its detection either by direct measurement of the singlet oxygen photons at 1270 nm (singlet oxygen luminescence) or indirectly using a fluorescent probe (right side).

Depending on the chemical structure of a photosensitizer there are two alternative kinds of pathways to generate reactive oxygen species (ROS) after light activation. A type I process involves the direct interaction of an excited photosensitizer with surrounding substrates to generate radicals or radical ions like hydroxyl radicals (HO^*∙*^) and superoxide anions (O_2_^∙−^) via charge transfer. Whereas in a type II mechanism the generation of singlet oxygen (^1^O_2_) usually takes place by direct energy transfer from the excited triplet state of the PS to molecular oxygen (see Figure 1). In both cases, the initially generated reactive oxygen species initiate further oxidized intermediates at the cell wall, cell membrane, on peptides, and lipids depending on the localization of the photosensitizer. 

The detection of reactive oxygen species is usually shown indirectly by deactivation of ROS using quenchers like sodium azide (type II), histidine, mannitol (type I), beta-carotene, and superoxide dismutase (type I). In biological systems, the lifetime of ROS can be very short (e.g., few *μ*s for singlet oxygen) [14]. Thus, the quencher/detection agents must be located directly at the site of ROS generation with a sufficient high concentration, which can be difficult and a source of ambiguous results. However, in microorganisms the transport of such quenchers is rather complicated and the quenchers may not reach the site of ROS generation. 

Increasingly sophisticated optical techniques have been developed and employed over the last years to both create and monitor ROS such as ^1^O_2_ in samples that range from liquid solutions to single living cells [15]. ^1^O_2_ is believed to play the major role in PDT/PDI. Therefore, the following text will focus on frequently used detection techniques for this type of ROS. Wu et al. have recently summarized methods of ^1^O_2_ detection [16], with focus on the recent technical advances. To investigate the chemistry of ^1^O_2_, several analytical tools are available to obtain information about the concentration, the spatial distribution, and the temporal behavior of ^1^O_2_ formation, including spectrophotometry, fluorimetry, and (chemi)luminometry. 

With respect to spectrophotometry, over the years, the design of appropriate probes for ^1^O_2_ has developed significantly. Up until the late 1990s many chemical ^1^O_2_ traps had been reported [17–21]. Of the trapping molecules used, 1,3-diphenylisobenzofuran (DPBF) has certainly been one of the favorites [22]. ^1^O_2_ rapidly and irreversibly reacts with DPBF to initially yield an endoperoxide which, in turn, evolves into other products that do not fluoresce and have absorption spectra different from that of DPBF. The development and use of DPBF-dependent techniques have recently played a key role in yielding useful information about ^1^O_2_  in a wide variety of systems [23–26]. 9,10-diphenylanthracene (DPA) was also a widely used chemical trap for ^1^O_2_ [27, 28]. In both cases, as well as for many other molecules, the fluorophore itself acts as a reactive moiety and changes its photophysical properties upon reaction with ^1^O_2_. The decrease in absorbance, due to the loss of conjugation, has been used for anthracene derivatives as a quantitative measure of the formation of the endoperoxide. However, because the detection of ^1^O_2_ was based on the measurement of absorbance changes, these probes are not highly sensitive.

**Figure 2:**
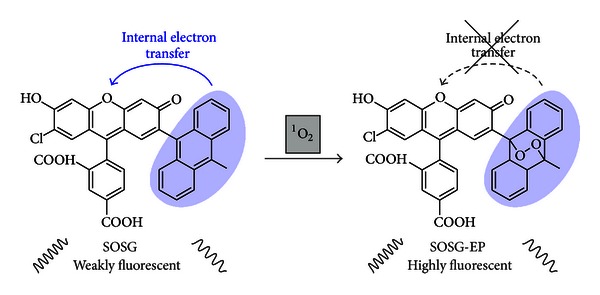
Formation of the endoperoxide of SOSG (singlet oxygen sensor green) upon reaction of SOSG with ^1^O_2_ as an indirect method for detecting ^1^O_2_. Prior to the reaction with ^1^O_2_, internal electron transfer (ET) quenches the fluorescence from the light-emitting chromophore. Upon reaction with ^1^O_2_ and the formation of the endoperoxide, electron transfer is precluded, and fluorescence is observed.

In 1999 Umezawa and coworkers designed and synthesized novel fluorometric probes for ^1^O_2_ based on fluorescence changes in order to improve the sensitivity [29]. They developed linked two-component systems called DPAXs, consisting of a fluorophore reporter portion and a ^1^O_2_-reactive anthracene moiety. The fluorophore part is based on fluorescein, which is widely used in cell biology for labeling and sensing [30]. In 2001, Tanaka et al. reported a rational design strategy for an optimal fluorescent probe for ^1^O_2_ also based on a fluorescein-anthracene combination called 9-[2-(3-carboxy-9,10-dimethyl)anthryl]-6-hydroxy-3*H*-xanthen-3-one (DMAX) [30]. The mechanism these probes are based on is photoinduced electron transfer (PeT). The experimental approach has been to devise a two-component system comprised of a trapping moiety coupled to a light emitting chromophore. Prior to the reaction with ^1^O_2_, emission from the chromophore is quenched by electron transfer from the adjacent trapping moiety. Upon reaction with ^1^O_2_, however, the resultant oxygen adduct is no longer an efficient intramolecular electron donor, and light emission readily occurs from the fluorescent moiety. Commercial vendors such as molecular probes also used this fluorescein-anthracene combination to develop singlet oxygen sensor green (SOSG, see Figure 2) [31]. 

Fluorescent probes are sensitive and can afford high spatial resolution via microscopic imaging [30]. The fluorescent properties of fluorescence probes such as fluorescence intensity, wavelength, quantum yield, or fluorescence lifetime can change upon reaction with ^1^O_2_. Two clear advantages of this indirect method to detect ^1^O_2_ exist: (1) the luminescence quantum efficiency of the optical probe is comparatively large (Φ_fl_ ~ 10^6^, Φ_ph_  (^1^O_2_) *≈* 1), and (2) emission occurs in the visible region of the spectrum where optical detectors are very efficient. However, for a number of reasons, caution must be exercised when using fluorescent probes such as SOSG. First, it was shown by Ragàs et al. that SOSG is able to generate ^1^O_2_ [32] and Gollmer et al. have shown that the immediate product of the reaction between SOSG and ^1^O_2_ is, itself, an efficient ^1^O_2_ photosensitizer [33]. Second, SOSG appears to efficiently bind to proteins which, in turn, can influence uptake by a cell as well as behavior in the cell [33]. Third, some fluorescent probes are not selective enough for one particular ROS. Recently, Nakamura et al. showed that SOSG reacted with other ROS such as O_2_^∙−^, hydrogen peroxide (H_2_O_2_) and HO^∙^ resulting in a small increase in the fluorescence response [34]. As such, incorrect use of fluorescent dyes can yield misleading data on yields of photosensitized ^1^O_2_  production and can also lead to photooxygenation-dependent adverse effects on the system being investigated.

Therefore, direct measurements are highly recommended to detect ROS like ^1^O_2_ via its luminescence at 1270 nm. In this case, photons that are emitted by the excited molecule itself are detected (Figure 1, right side). An advantage is that there is no need for any additional substances, which might be either toxic or too large for transportation in tissue. The time-resolved detection technique has been used for (1) the identification of ^1^O_2_, (2) measurement of quantum yields of ^1^O_2_ production in photosensitized processes, Φ_Δ_, and (3) the determination of rate constants for the interaction of ^1^O_2_ with substrates.

The ^1^O_2_ signal depends on many parameters that are expressed in the following formula:

(1) [O12](t)=[T1]0kT1Δ[O32]kT−kd(e−kdt−e−kTt),

where [*T*_1_]_0_ is the concentration of the photosensitizer molecules in the excited *T*_1_ state, *k*_*T*_1_∆_ is the rate constant for deactivation of the *T*_1_ state by ^1^O_2_, [^3^O_2_] is the concentration of molecular oxygen in its ground state, *k*_*T*_ is the rate constant for all channels of *T*_1_ deactivation, and *k*_*d*_ is the rate constant for all channels of ^1^O_2_ deactivation.

The time dependence of the ^1^O_2_ signal is used to better understand the interaction of ^1^O_2_ with its environment. It tells one about the kinetics of the production and the decay of ^1^O_2_ in different environments ranging from polymer systems to single living cells. These direct measurements can reflect the complex and dynamic morphology of a cell [35–39].

The ^1^O_2_ luminescence quantum efficiency (Φ_Ph_) is given by (2):

(2) $$\begin{array}{c} \Phi_{\text{Ph}}=\Phi_{\Delta }k_{r}\tau_{\Delta }, \end{array}$$

where Φ_Δ_ represents the quantum yield of ^1^O_2_ formation, *k*_*r*_ the radiative rate constant for the transition ^1^O_2_ → ^3^O_2_, and *τ*_Δ_ the lifetime of ^1^O_2_.

However, despite the wide spread use of the direct 1270 nm detection, it has limitations because of the low ^1^O_2_ luminescence (the quantum efficiency is about 10^−5^–10^−7^ depending on its environment) [40] and low signal-to-noise ratios, which makes the measurement of the ^1^O_2_ signal a nontrivial task and limits the effectiveness of this technique for many applications especially in the biological field. In the presence of molecules that can physically quench or chemically react with ^1^O_2_, such as water or proteins, for example, in a biological cell, *τ*_Δ_ will decrease and Φ_Ph_ will also decrease, resulting in a low signal-to-noise ratio. However, for the measurement of the luminescence signal a very sensitive detection system is available. With respect to ^1^O_2_ detection, the use of the 1270 nm ^1^O_2_ → ^3^O_2_ luminescence in both time and spatially resolved experiments has, without doubt, been the most beneficial and informative tool. Several research groups were able to detect singlet oxygen luminescence in lipids and even in living cells/microorganisms after incubation with an exogenous photosensitizer and an optical excitation at different wavelengths [41–44].

### 2.3. Assessment of Radical Formation

In the type I mechanism of the photodynamic principle hydrogen-atom abstraction or direct electron-transfer reactions occur between the light excited state of the photosensitizer and a substrate that can be either a biological structure (e.g., lipids, proteins, amino acids, or DNA), a solvent, or an inanimate surface to yield free radicals or radical ions like superoxide radicals, hydroxyl radical, or peroxyl radical [45]. The main methods for detection of type I-generated radicals include scavenging molecules such mannitol, histidine, and N,N-dimethyl-p-phenylenediamine (DMPD), as well as the total radical-trapping antioxidant parameter method (TRAP) or the oxygen-radical absorbance capacity (ORAC) method. At present, there is a need for more specific assays due to the lower specificity of these scavenging methods. Overall, type I reactions become more important at low oxygen concentrations like inside of biofilms or in more polar environments [46].

### 2.4. Determination of the Photostability/Bleaching

Due to light exposure of a photosensitizer, the absorption and fluorescence properties of the photosensitizer itself can change, which indicates that photodegradation and/or photoproduct formation appeared which results in a decreased photostability [47, 48]. Such changes can be evidenced by the appearance of new absorption bands within the specific absorption spectra of the photosensitizer [49]. Degradation can be monitored by the decrease of maxima absorption peak of the photosensitizer. Changes of the characteristic absorption spectra of a given photosensitizer depend on the wavelength of the illumination light: shorter wavelengths are more effective than longer wavelengths [50]. If the photosensitizer is bleached too rapidly, either successful inactivation of microorganisms or tumor destruction will not be completed once the minimal inhibitory concentration of the nondegraded photosensitizer in the infected/tumor tissue is deceeded upon illumination [51]. On the other hand, photobleaching can be an advantage regarding avoiding an overall skin photosensitivity which is one of the main side effects in patients treated with PDT [1, 52]. Furthermore, it is unalterable to assure the nontoxicity of photodegraded products of the photosensitizer. Overall knowledge of photodegradation as well as effects of photoproducts generated upon illumination is important to develop an appropriate dosimetry for each photosensitizer in antimicrobial photodynamic inactivation or in antitumor PDT [53].

### 2.5. Positive Charge and Molecular Weight of a Given Photosensitizer

A must-have of a successful PS for PDI is a positive charge, because bacteria are charged negatively due to their cell wall composition and *meso*-substituted, but negatively charged, porphyrins have not shown toxicity against Gram(−) bacteria [54, 55]. Furthermore, hydrophilic compounds (less than 600–700 Da, e.g., for *E. coli*) can diffuse only through the outer membrane via porins which act as a very effective permeability barrier, making Gram(−) bacteria less susceptible as Gram(+) [56]. Therefore, porphyrin-based photosensitizers like TMPyP with a molecular weight higher than 500 Da cannot diffuse through these porin channels. As a consequence, ROS can be generated only at the cell wall area of Gram(−) bacteria. Another important observation that has been made about positive-charged cationic antimicrobial photosensitizers concerns their selectivity for microbial cells compared to host mammalian cells [57]. It is thought that cationic molecules are only slowly taken up by host cells by the process of endocytosis, while their uptake into bacteria is relatively rapid. If illumination is performed within short intervals after PS application (minutes) the PDT-mediated damage to host tissue will be minimized. 

## 3. PS Uptake Kinetics, Dark Cytotoxicity, and Intracellular Localization in Tumor Cells

This section describes experimental approaches for initial tumor cell-based characterization of new PS including cellular pharmacodynamics, cytotoxic effects of the PS in the absence of light, and the intracellular localization of the PS. Finally, the assessment of the penetration depth in an *ex vivo* porcine skin model is described. 

### 3.1. PS Uptake/Release Kinetics

Measurement of the uptake of a new PS drug into cancer cells provides information on the (kinetics of) interaction and membrane transport characteristics of the drug and enables a first rough estimation of the drug-to-light interval—as a basis for verification in the subsequent preclinical validation of the PS. The methodological approaches described here make use of the inherent fluorescence properties of the PS and allow for either absolute quantification of the drug amount bound respective taken up by cells or a relative quantification mainly, for example, for estimation of time-dependent course of PS uptake.

By the nature of the assay format, experiments using microplates with 96 wells as the most commonly employed format allow for time-efficient and multiparametric testing of several variables possibly influencing the uptake characteristics. A first approach involves incubation of cancer cells cultured in 96-well microplates, followed by a fixed incubation period (e.g., 10–24 hrs) with a dilution series of the PS in the appropriate cell culture medium and subsequent determination of the PS-related fluorescence signal. The simplest procedure involves washing the cell cultures after the incubation and lysis of the cells by detergents such as SDS (sodium dodecyl sulphate) followed by fluorescence measurement in a microplate fluorimeter. Control experiments should be performed to exclude altered fluorescence characteristics of the PS in the presence of such cell lysis reagents. Besides the plasma membrane permeability of the given PS, several additional factors may influence the cellular uptake including interaction of the PS with (i) constituents of fetal calf/bovine serum (FCS/FBS; [58, 59]) and with (ii) the cell culture plastic material [60, 61]. The first parameter can be assessed by using appropriate concentrations of FCS ranging from zero to the standard concentration used for routine cultivation of the respective cell type (e.g., 5%–15% v/v FCS for numerous cancer cell lines). Such data are important as similar serum constituents are present in human blood plasma which may influence the tissue distribution of PS drugs after systemic administration. The second parameter may significantly influence the results obtained from the described simple incubation-lysis-measurement approach as especially lipophilic PSs may attach to a considerable amount to the cell culture plastic surface [60]. As we have demonstrated in a recent study ([61], this issue), the surface-adhered PS in microplates without cells can even exert considerable phototoxic effects after addition of PS-free cells in a range similar to the usual protocol where cells are seeded first and the PS is added subsequently. Therefore, appropriate control experiments and controls samples need to be included to estimate the amount of PS which is bound by the microplate plastic and which might significantly contribute to the PS fluorescence signal measured after PS incubation and direct lysis of the cells. In case of a rather lipophilic PS, such false-positive fluorescence signals can be avoided by detaching the cells from the culture receptacle (e.g., trypsinization or EDTA treatment), transfer to new tubes/wells followed by cell lysis and fluorescence detection. Regardless of whether cell-bound PS fluorescence is measured directly in the microplate wells or after detachment and transfer, this approach can be designed to allow for absolute quantification of the amount of cell-bound PS if appropriate cell-free PS dilutions series are simultaneously measured. 

An alternative approach is based on fluorescence-activated cell sorting (FACS) analyses based on single-cell analysis of the cellular fluorescence in a flow cytometer. As such protocols involve detachment of cells prior to analysis, no interference with the measured signal from PS molecules adhered to the plastic material is expected. On the other hand, this approach is only suitable for relative quantification and each sample usually requires manual work (e.g., for cell detachment, transfer, washing steps) implicating a reduced number of processable samples compared to the microplate format.

Both experimental approaches can be used to characterize the dose-dependent PS uptake by cancer cells by identification of the range of PS concentrations which result in a measurable PS fluorescence—that is, the minimum concentration as well as, a concentration threshold above which a saturation of PS bound/taken up by the cancer cells occurs. With both methods, the influence of the presence of serum constituents as well as for example, the influence of the chemical PS formulation can be assessed. Subsequently, the time-dependent uptake studies can be performed to investigate the kinetics of PS uptake into cells. As mentioned previously these data may give first information on the appropriate drug-to-light interval and allow for definition of an incubation period for subsequent *in vivo* experimentation. Particularly for time-dependent experiments involving incubation times in the range of several hours up to days it is important to correct for the cell number (biomass) present at the individual time points. This can be achieved, for example, by measurement of the total protein content (e.g., colorimetric assays such as Bradford, bicinchoninic acid assays) of the cell sample as a surrogate parameter for the actual cell number. Clearly, in highly proliferating cell types, the cell number may influence the amount of PS bound/taken up by the individual cell—especially at low PS concentrations. 

The release of PS by cells—that is, transport of the PS molecules out of the cell—is an important parameter as it partly determines the period of photosensitivity in the clinical application; that is, a PS drug which is rather rapidly exported from cells is likely to bring about a reduced time period necessary for the patient to readapt to normal daylight conditions. PS release experiments can be performed using both of the previously mentioned approaches and additional parameters such as the presence of FCS in the culture medium can be tested for their influence on the release kinetics. In our hands, the FACS-based approach seems more reliable for this purpose, as its single-cell measurement may allow for more accurate determination of the residual cell-bound PS (resp., the amount of PS released from cells) than if it is summarily measured in lysed cells. After the PS incubation period, PS release experiments involve careful washing of the cell cultures to remove unbound PS molecules and subsequent incubation in PS-free cell culture medium. In general, time periods ranging from, for example, 10 to 24/48 hours should serve as valid starting points for analysis of the PS release. 

### 3.2. PS Dark Toxicity

The general requirements for an optimal photosensitizing agent include low dark toxicity, that is, negligible cytotoxicity in the absence of light. This ensures the validity of the dual-specificity ideal of antitumor PDT, namely that both via tumor cell (semi)selective enrichment of the PS and confinement of the illuminated area by appropriate design of the light source, the cytotoxic action of PDT is limited to the cancerous tissue while sparing adjacent healthy cells [62]. Experimental assessment of the dark toxicity involves incubation of the cells with a PS dilution series initially according to the incubation parameters established in the PS uptake experiments described in Section 3.1. Probably, the establishment of optimal parameters (e.g., incubation time, PS concentration, media composition, and cell density) may require that experiments on PS uptake characteristics and dark toxicity are performed in parallel. In general, a large array of appropriate assays is available for viability analyses in *in vitro* cell culture samples. These assays make use of either measurement of (i) metabolic parameters (activity of metabolic enzyme) as a surrogate readout for the cell's viability, (ii) biochemical and morphological changes during apoptosis, (iii) proliferation rates of cells, and/or (iv) viable cell number employing specific membrane-impermeable dyes to exclude dead (leaky) cells. For determination of dark toxicity of a PS in a given cell type, usually only the overall effect on viability or proliferation is interesting. More detailed analysis of the modes of cell death is rather important for the investigation of the light-induced effects of the PS (see Section 4). Anticipatorily, these tests are listed here in Table 1 including a superficial appraisal of their various strengths. 

For the particular assessment of dark toxicity, classical viability tests based on measurement of the activity of metabolic enzymes may be most efficient since these tests can be performed in microplates implicating the possibility of multiparametric testing including technical replicates for each sample. 

### 3.3. Intracellular PS Localization

Following establishment of the overall PS uptake characteristics and the PS's dark toxicity, a subsequent experimental step involves the determination of the intracellular localization and enrichment of the photosensitizing drug. Again, for this purpose, the inherent fluorescent properties of the PS are used.

Provided the microscope setup is equipped with the appropriate filter sets, first superficial information on the intracellular distribution can be obtained from conventional fluorescence microscopy. With this approach, general statements such as preferential localization in the cytoplasm, plasma membrane, or the perinuclear region can be obtained. For more detailed analysis, the use of specific organelle-localizing dyes (“organelle trackers”) is recommended.  Table 2 provides a list (not complete) of fluorescence dyes that might be used for analysis of colocalization with the PS investigated. The choice of a particular dye depends on its fluorescence spectrum which should not overlap with the emission wavelength of the PS. Particularly, if the localization of the PS is not confined to one clearly identifiable structure, the use of confocal fluorescence microscopy which provides increased spatial resolution may be helpful. An alternative approach for quantification of the PS's intracellular localization could involve organelle-specific fractionation, for example, by centrifugation techniques and analysis of the organelle-bound PS fluorescence—this approach is more time-consuming and may require more extensive optimization for the particular cell type. 

### 3.4. Assessment of the Penetration Depth of the PS in Porcine Skin

After a first positive prescreening of new developed photosensitizers with photodynamic activity against microorganisms in suspension *in vitro*, the next challenge in antimicrobial photodynamic inactivation is to find appropriate parameters (e.g., light dose and incubation time) to inactivate relevant key pathogens without harming surrounding tissue *in vivo/ex vivo*. Therefore penetration and localization of the given photosensitizers must be investigated using an *ex vivo* skin model. Recently it could be shown that an *ex vivo* porcine skin model can be used, because it is proposed as a good test model for human skin based on many similarities regarding physiological, histological, and permeability properties [63]. Restriction of a photosensitizer to the stratum corneum without accumulation in deeper parts of the epidermis or dermis might be useful regarding a successful decolonization of pathogens on intact skin [64]. Recently it could be shown that localization of the photosensitizer TMPyP in a water-ethanol formulation was restricted to the skin surface only [64]. However agents with a molecular weight of >500 Da exhibit a low permeability through the stratum corneum. The molecular weight of TMPyP is 682.2 g·mol^−1^ (without counterions). Therefore the molecular weight of drugs which are used in transdermal drug-delivery systems is well below <500 Da [65]. To enhance penetration through the stratum corneum various formulations are available that contain supplements (DMSO, alcohol, pyrrolidones) which exhibit penetration enhancing activities [66]. Overall the main targets are superficial and localized infections. These areas are readily accessible for the topical application of PS and light, neither harming the surrounding tissue nor disturbing the resident microbial flora.

## 4. Photodynamic Action in Tumor Cells

This section describes experimental approaches for characterization of the cytotoxic action induced by light including analysis of overall viability, IC_50_ values, and, specifically, the discrimination of the cell death modes induced by PDT. 

### 4.1. Analysis of Tumor Cell Viability Changes after PDT

After having optimized the incubation parameters (concentration, incubation time, and media composition) as described in Sections 3.1 and 3.2, one can proceed in analysis of the photoinduced cytotoxic effects of a new PS. Using adherent cancer cells (cell lines) the fastest approach is to use a microplate assay based on the activity of metabolic enzymes such as MTT or the resazurin assay, both of which are quick, cheap in terms of reagents, and easily established (see Table 1 for an overview). Similar to such assays, determination of intracellular ATP gives a reliable estimation of the amount of viable cells after a cytotoxic treatment as the intracellular concentration of this metabolite (as is the activity of core metabolic enzymes) is assumed to be held in a tight (millimolar) range in viable cells. Therefore, the overall amount of ATP or the enzyme activity in a population of cells is supposed to represent the overall viable cell number. Important to keep in mind is the fact that cells undergoing apoptosis (active cell death) maintain considerable levels of metabolic activity respective ATP in order to perform the energy-requiring steps during the apoptotic cascade [67–72]. Therefore, for all of the mentioned assays, the time point to perform the test should be chosen in a way so that apoptotic cells do no longer contribute to the assay readout. This might include establishment for each individual cell line in order to determine the time point after treatment where apoptotic cells have finished the cell death program and have undergone secondary necrosis. This time period may be in the range of 24–48 hrs after treatment for most cancer cell lines.

**Figure 3:**
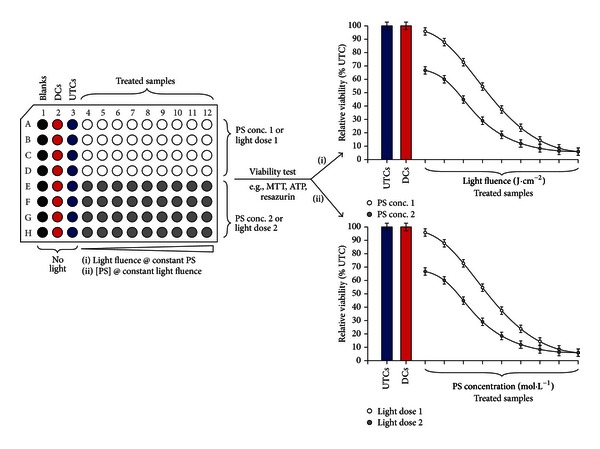
Experimental variants for analysis of overall tumor cell viability following PDT. Under constant PS conditions or constant light fluence, the light fluence or the PS concentration can be varied to obtain initial information on the phototoxic effects of a particular PS. DC: dark controls (PS without light); PS: photosensitizer; UTC: untreated control (no PS, no light).

Besides PDT-treated cell samples, each particular experiment on overall cell viability should include the following control samples—again most easily to be realized in the (96-well) microplate format: untreated control (UTC), dark control (DC), light-only control (LOC), the treated samples, and appropriate blank wells required for the assay's blank subtraction. In our experience, at least triplicate wells should be included for each of the listed sample types. Treated samples are incubated with the PS (as established in Section 3.1 to result in a measurable cellular enrichment of the PS). As illustrated in Figure 3, subsequent illumination conditions can be designed in two ways to get an overall impression on the viability changes following PDT treatment: (i) a constant PS concentration is employed for all treated samples accompanied by illumination with different light fluences (J·cm^−2^) or (ii) incubation with different concentrations of the PS (i.e., a dilution series) followed by illumination with a constant light fluence. Particularly when different light fluences are applied to individual rows of the microplate wells (as in Figure 3; approach (i)) microplates with clear well bottoms and black walls should be employed to avoid activation light crosstalk between the rows of wells during illumination. 

Similar to determination of the dark cytotoxicity of the PS, comprehensive characterization of the PDT *in vitro* model system should address—or exclude, in most cases—possible cytotoxic effects of the illumination itself. For this purpose, cells are seeded and incubated in the same medium as usually used for PS incubation but without the PS, followed by illumination with different light fluences. As mentioned, for most applications such a control experiment is to rule out possible effects of the illumination itself on the viability or proliferation rate of the cells. 

All the mentioned experimental approaches should be accompanied prior to the particular assay by routine control observation in a conventional light microscope. In most cell lines, cytotoxic effects can be readily identified at this level by observation of rounding of cells (apoptosis, probably including classical apoptotic bodies) and detachment of cells (apoptosis and/or necrosis at later time points). Such visual control may help interpretation of the results gained from assays measuring metabolic enzymes or metabolites as a surrogate parameter of cell viability and help rule out false-positively or -negatively high/low signals. 

### 4.2. Calculation of the IC_50_ Values

The mentioned analyses of dark toxicity and light-induced cytotoxicity (using different PS concentrations and a constant light dose) can be used to calculate a modified IC_50_ value. This parameter is usually referred to as the half-maximal inhibitory concentration of an inhibitor in, for example, enzyme inhibition experiments. In the context of PDT, the IC_50_ value is calculated by division of the concentration required for 50% cell killing in the dark (lethal dose (LD_50,dark_)) and the concentration required for 50% cell killing following illumination of PS-incubated cell samples (LD_50,PDT_) [74]. The IC_50_ value thus measures the relation between the cytotoxic effects of the PS in the dark and following photoactivation; a higher IC_50_ is indicative of a low dark toxicity or a particular high cytotoxic efficiency after illumination [75]. By its nature, use of this parameter makes only sense for direct comparison between two or more PSs in the same cell model and under comparable illumination conditions [75, 76]. 

### 4.3. Analysis of the Cell Death Mode

**Figure 4:**
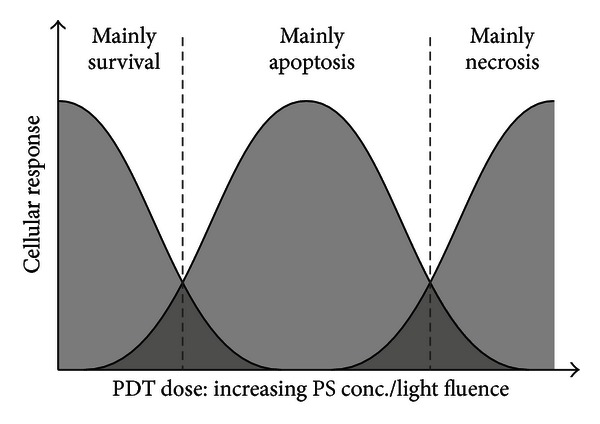
Dose-dependent transition between cellular responses following PDT. Abbreviations: PS, photosensitizer. Modified from [73].

Further in-depth analysis of the cytotoxic action of a PS following illumination involves the discrimination between essentially three modes of cytotoxicity, that is, inhibition of proliferation, induction of apoptosis, and, third, induction of necrotic cell death. Very low PDT doses may also cause increased cell proliferation resulting in increased cell viability signals and/or cell numbers. As discussed recently, the mode of action of PDT can usually cause all the mentioned effects—in a dose-dependent manner as illustrated in Figure 4. This is in contrast to chemotherapeutic agents or radiation which preferentially causes apoptosis as the underlying cytotoxic effect [1, 79, 90].

**Figure 5:**
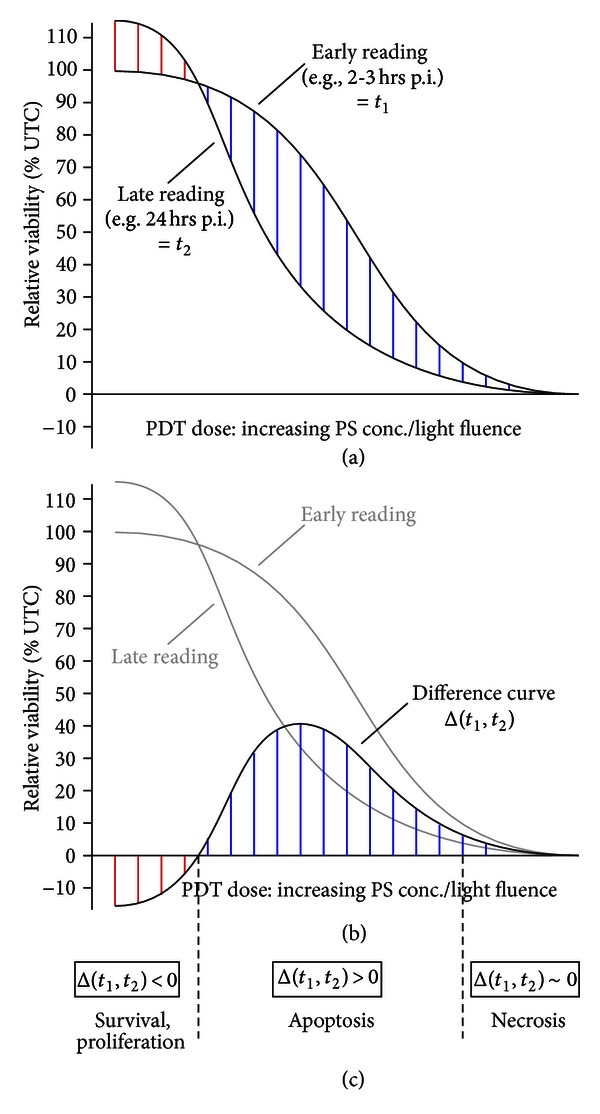
Discrimination of the cellular responses towards PDT. Measurement of the overall viability at early versus late time points following illumination (a) allows for calculation of a difference curve (b) and estimation of the dose ranges which predominantly induce cellular survival, apoptosis, and necrosis (c). PS: photosensitizer. For details on interpretation see text.

For rapid and initial analysis of the cell's response in terms of proliferation, apoptosis, and necrosis, our group has developed a simple and versatile assay based on microplate assays analyzing metabolic enzymes [67, 70]. This procedure makes use of the fact that cells undergoing apoptosis (i.e., active cell death) require functioning energy supply in terms of intracellular ATP accompanied by approximately normal activity of (catabolic) pathways whose enzymes are those measured in the MTT test, for example. As shown in Figure 5, this approach employs standard viability tests based on metabolic surrogate parameters (e.g., MTT, ATP, resazurin) and involves measurement at two different time points following PDT treatment: a first measurement is taken at an “early” time point where cells undergoing apoptosis still retain their metabolic activity. A second reading is taken at a “late” time point where apoptosis has been completed and these cells have converted to secondary necrosis due to the absence of phagocytizing cells in the cell culture setting. In contrast to apoptotic cells which maintain their metabolic activity until the late steps of the apoptotic program, necrotic cells are characterized by a rapid breakdown of the plasma membrane integrity, metabolic hemodynamics, and a leakage of intracellular material in the extracellular space [91]. 

As shown in Figure 5, the different signals between early and late readings can be used for a first discrimination between induction of proliferation, apoptosis, or necrosis in the *in vitro* setting. This approach clearly works with sum signals; therefore, mixed populations of, for example, apoptotic and necrotic cells cannot be quantified in absolute terms. However, the 96-well microplate format—on the other hand—allows for rapid testing of, for example, ten different treatment conditions. This assay variant has been successfully used in previous publications with either the MTT assay [67, 70] or the fluorescent resazurin assay [82].

Another simple test for discrimination of whether a reduced viability signal is caused by direct cytotoxicity (apoptosis or necrosis) or by inhibition of proliferation also employs metabolic viability tests such as the MTT assay. For this purpose multiple readings at different time points following illumination are performed. The viability signals obtained at each time point are related to the initial (*t* = 0 hrs) value and the resulting temporal dynamics of the signal for each treatment condition can be evaluated as follows: a decrease below the initial value can be interpreted as a direct cytotoxic effect as the absolute viability signal decreases. A constant viability signal (in the range of the initial value) indicates inhibition of proliferation, whereas a signal increasing relative to the initial value (similar to untreated controls in most cases) indicates proliferation. Clearly, as a sum measurement this test design cannot discriminate between the modes of cytotoxicity in absolute terms (i.e., on the single-cell level). However, it may assist in the interpretation in a situation where the endpoint viability measurement (e.g., 24 hrs p.i.) indicates a viability signal smaller than the untreated controls since this reduction could be solely attributed to growth inhibition without any apoptosis/necrosis induction, that is, direct cytotoxicity. Assays directly measuring the proliferation rate are classically based on DNA incorporation of nucleotide analogues such as ^3^H-thymidine or bromodeoxyuridine (BrdU). Incorporation of the first can be measured via scintigraphy whereas the latter is detected by BrdU-specific immunostaining. Both methods allow direct assessment of the proliferation rate of cells but should be accompanied by the mentioned viability tests for unequivocal interpretation. 

After having superficially determined how the cell population responds to a photodynamic treatment (survival/proliferation, direct cytotoxicity/reduced viability), one may proceed to in-depth characterization of the specific mode of cell death in case of a cytotoxic PDT regimen. This might be relevant to elucidate the detailed mechanism of a particular PS or PDT regimen and, on the other hand, might have implications for overall therapeutic effect by induction of diverse immune system-related responses [92, 93]. In this brief discussion we focus on the classical ways of cell demise—excluding autophagy which is also considered in recent reports to contribute to PDT-induced cytotoxicity [94] (for an methodological overview see [95]).  Table 3 lists the most common methods and assays to address whether cells and cell populations undergo apoptosis (active/“programmed” cell death) and/or necrosis (passive cell death) (further reading [96, 97]). As commented in Table 3, the variety of methods differs with respect to their quantitative or semiquantitative results, that is, whether a percentage of cells undergoing apoptosis can be determined using the particular method. Furthermore, the assays differ with respect to price as well as prerequisites regarding instrumentation and time required. Furthermore, some of the listed approaches may depend on whether the cell (or cell line) studied shows the morphological/biochemical feature addressed by the particular test. A comprehensive weighting of the various methods is beyond the scope of this section—useful advice in our opinion includes the following aspects: (i) appropriate—probably non-PDT-treated-control samples (i.e., 100% apoptotic/necrotic cells) help to validate the method and make the results obtained for PDT-treated samples more reliable and expressive, (ii) whenever possible, a population-based method should be accompanied by single-cell analysis(es) to gain information about the cell portion affected, (iii) all indirect (non-microscopy-based) assays should be accompanied by simple (phase contrast) light microscopy to allow comparison with the sometimes quite obvious overall cellular responses, (iv) the timing when to use individual methods may need optimization for each cell model (and treatment protocol) as some of the cellular/biochemical events listed in Table 3 occur early versus rather late following the PDT treatment, and (v) PDT treatments may cause mixed population consisting of both apoptotic and necrotic subpopulation of cells in a given sample ([73], see also Figure 4). The methods listed in Table 3 comprise assays specifically focusing on *in vitro* experimentation using cells (cell lines) in culture; for specific methods to investigate the occurrence and extent of apoptosis/necrosis *in situ* (tissue sections) the reader is kindly referred to recent methodological overviews [98, 99]. 

## 5. Characterization for Photodynamic Inactivation of Eukaryotic and Prokaryotic Microorganisms

Ideally, a wide spectrum of antimicrobial action on bacteria, fungi (yeasts), and protozoa should be achieved with a given PS/PDI protocol. The primary readouts should focus first on photodynamic efficacy in suspensions, followed by biofilm inactivation (monospecies and polyspecies) *in vitro*. Later on *ex vivo* and animal studies should be considered to demonstrate a photodynamic killing efficacy of ≥3 log_10_ steps (≥99.9% reduction of viable microorganisms). Such a reduction of viable microorganisms must be achieved to state that an antimicrobial effect is possible. Furthermore, the efficacy should be independent of the antibiotic/antifungal resistance pattern of the investigated microbial strains. From this point of view a selection of photodynamic-resistant microorganisms should be absent after multiple sublethal treatments conditions. Due to the regulatory affairs to get approval by the FDA or the European Health Authorities, mutagenicity must be excluded. Appropriate formulations must be developed allowing an easy and specific delivery of the given photosensitizer to the infected area. 

### 5.1. Assessment of Cytotoxicity in the Dark and Phototoxicity Based on CFU Counting

The American Society of Microbiology has decreed that for any technique to be called “antibacterial” or “antimicrobial” at the very least 3 log_10_ of CFU (99.9%) need to be killed. Furthermore based on the guideline for hand hygiene in health-care settings a minimum of 5 log_10_ reduction of viable counts of microorganisms must be achieved for a successful disinfection [100]. Survival of viable bacteria must be determined by the colony-forming assay. After overnight incubation, colonies are counted and viable pathogen concentration is expressed as CFU/mL using a logarithmic scale. Furthermore, no cytotoxic effects in the dark of both the given photosensitizer itself and of the possible photoproducts formed after illumination should be demonstrated either against the pathogen itself or the eukaryotic cells. 

### 5.2. Addition of Cell Wall-Permeabilizing Agents

In case that the given photosensitizer is not efficient enough (less than 3 log_10_ steps of CFU/mL reduction) to kill relevant pathogens upon illumination addition of cell wall-permeabilizing agents might be useful to enhance the photodynamic efficacy. From a clinical point of view metal chelators like EDTA might be useful to cause a disorganization of lipid structures increasing the permeability of the outer membrane of Gram(−) bacteria [54, 101]. EDTA solutions might be useful, because it is well established in dentistry as it has been commonly used as a detergent for the removal of smear layers. Another permeabilizing agent is polymyxin B nonapeptide which has demonstrated a porphyrin-based photodynamic enhancement [55].

### 5.3. Determination of the Efficiency towards Bacterial Biofilms

The natural behavior of microorganisms is to grow as a biofilm rather than as free-floating cells. It is generally accepted that biofilms represent the leading cause of microbial infections. One of the main consequences of the biofilm mode of growth is the increased resistance to antimicrobial therapy, resulting in recurrent or persistent infections leading to treatment failure. Therefore, biofilms are up to 100-fold more resistant against any antimicrobial treatment modality as compared to their planktonic counterpart [102]. From this point of view it is necessary to evaluate additionally the photodynamic efficacy against biofilm growing pathogens of any positive preselected photosensitizer which has demonstrated photodynamic killing efficacy against microorganisms in suspension.

## 6. Conclusion

**Figure 6:**
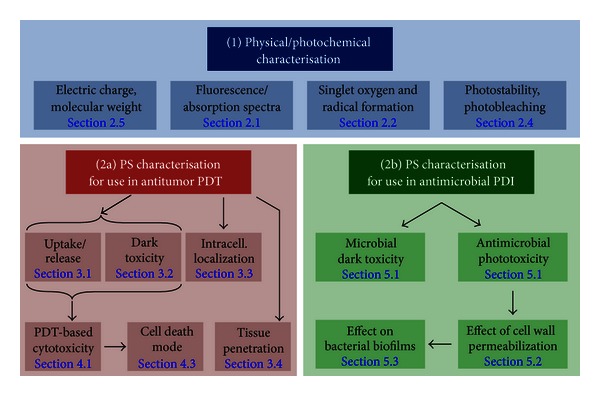
Flow chart for basic characterization of novel photosensitizers for PDT and/or PDI applications. This diagram provides a suggested stepwise procedure for basic and *in vitro* characterization of novel PS molecules involving the most important physical, photochemical and cellular characteristics. The respective sections within this paper are provided.

Taken together, the previously mentioned experimental approaches are suited to provide comprehensive information on the potential use of a particular—and probably newly developed—PS agent in the frame of PDT or PDI. As several aspects determine the possibilities of use in the two major branches of photodynamic applications (see the aforementioned for details), we suggest a stepwise characterization of the most important physical and photochemical/-dynamic features of a new PS in order to determine the possible fields of therapeutic applications. Such a step-by-step approach is depicted in Figure 6 (with reference to the chapters within this paper) providing an interdisciplinary straight forward strategy for comprehensive characterization of photosensitizing compounds. Early and unequivocal identification of the various strengths and weaknesses of an individual agent may help deciding for which particular clinical application the particular drug is worth further establishing.

*In vitro* research represents the initial step in the biological characterization of a new PS for its application in PDT or PDI. Conclusions drawn from cell culture experiments are always difficult to directly transfer to the *in vivo* situation and may not allow for a very precise prediction of clinical applications of a given substance. However, these experiments may suggest possible targets and provide first evidence on practicable PDT/PDI treatment protocols. Up to date, no standard strategy for the basic *in vitro* investigations of PS existed. Therefore, this tutorial, which is based on the authors' experience in PDT and PDI, can serve as a guide for researchers who are involved in preclinical PS testing or plan to contribute to such research efforts. Special aspects of the experimental categorization of a novel PS, such as, for example, the possible interference of the PS with fluorochromes employed in cytological assays or the light sensitivity of the PS, have to be considered by an experimenter when performing a spectral analysis, the determination of levels of the phototoxic agents (ROS) including singlet oxygen, drug and light dose finding, intracellular localization, or assessment of the mode of cell death predominant at a given PDT protocol and are discussed in this paper. As outlined before, PDI research using microorganisms as model systems has to take into account the special nature of these pathogens. For example, the high growth rate of bacteria and yeast reasons the requirement of assays which allow for viability tests covering more than three orders of magnitude, which excludes classical colorimetric assays. Furthermore, as discussed in the previous chapters, the presence of cell wall composition and the ability to form biofilms require alternative PS and therapeutic procedures for successful photokilling when compared to cancer cells.

Until now localized infections of the skin, wounds, infections of the oral cavity, infections related to periodontitis, and endodontitis as well as infection of the middle ear are especially suitable for PDI treatment, because they are relatively accessible for PS application and illumination [103]. Overall, PDI might either be substitute standard antimicrobial therapy or act as an additional approach in the future.

Concluding, we hope that this tutorial will motivate researchers of all disciplines to get involved in photodynamic therapy and photodynamic inactivation and thereby help to further expand the convincing benefits of photodynamic procedures to new fields of applications. 

## Figures and Tables

**Table 1:** Tests for cell viability and cell death modes.

Assay type	Test	Measured parameter	Signal			Instrument	Microplate^a^	References^b^
			ABS	FI	Lumi			
Metabolic enzyme(s)	MTT, XTT, WST	Mitochondrial enzymes	X			Microplate reader	Yes	[77–79]
	Resazurin	Cellular dehydrogenase enzymes and cytochromes		X		Microplate reader	Yes	[80]

Metabolites	ATP	Intracellular ATP			X	Microplate reader	Yes	[81]

Apoptotic changes	Caspase activation	Apoptosis-specific proteases	X	X	X	Western blot	No	[77]
						Microplate reader	Yes	[75]
	Nuclear fragmentation	Chromatin condensation and fragmentation		X		Fluorescence microscope	Yes	[82]
						Flow cytometer	No	[75]
	DNA ladder	DNA cleavage resulting in multiples of 180 Bp		X		Gel electrophoresis	No	[83]
	Membrane blebbing	Characteristic apoptotic bodies	X			Phase contrast microscope	Yes	[67]
	PARP cleavage	poly-ADP ribose polymerase cleavage		X	X	Western blot	No	[77]
	Annexin V	Membrane externalization of Annexin V		X		Flow cytometer	No	[77]
						Fluorescence microscope	Yes	[77]

	Cyt-c release	Mitochondrial cyt-c release		X	X	Western blot	No	[77]

	ΔΨ	Mitochondrial membrane potential breakdown		X		Flow cytometer	No	[67, 84]
						Fluorescence microscope	Yes	[85]

Cell proliferation	^ 3^H thymidine	DNA incorporation of ^3^H thymidine/BrdU				Scintillation counter	Yes	[86]
	BrdU		X			Microplate reader	Yes	[87, 88]

Cell number		Direct cell number		X		Flow cytometer	No	

ABS: absorbance; ATP: adenosine-5′-triphosphate; BrdU: 5-bromo-2′-deoxyuridine; caspase: cysteine-dependent aspartate-directed proteases; FI: fluorescence intensity; MTT: 3-(4,5-Dimethylthiazol-2-yl)-2,5-diphenyltetrazolium bromide; Lumi: luminescence; PARP: poly(ADP-ribose) polymerase; WST: water-soluble tetrazolium salt; XTT: 2,3-bis-(2-methoxy-4-nitro-5-sulfophenyl)-2H-tetrazolium-5-carboxanilide.

^a^Assay suitable for use with microplates (yes/no).

^b^Methodological references or exemplary studies using the respective test in the context of *in vitro* PDT.

**Table 2:** Fluorescent probes for cellular organelle counterstaining [89].

Organelle/cell structure	Fluorescent dye(s)
Mitochondria	TMRM, TMRE, rhodamine 123, tetramethylrosamine, mitotrackers, nonyl acridine orange, carbocyanines, dual-emission dyes (JC-1, JC-9)
Endoplasmic Reticulum	3,3-dihexyloxacarbocyanine iodide [DiOC6(3)], ER-Tracker
Nucleus	DAPI; Hoechst-33342, propidium iodide, SYTO dyes
Cytoplasm	Calcein AM
Golgi apparatus	Fluorescent labeled lectins
Lysosomes	LysoTracker
Cell membrane	CellTracker

DAPI: 4′,6-diamidino-2-phenylindole; TMRE: tetramethylrhodamine ethyl ester; TMRM: tetramethylrhodamine methyl ester.

**Table 3:** Cell-based assays for discrimination and quantification of cell death modes.

Cellular/biochemical event	Method^a^	Assay platform	Comment^b^
DNA degradation	(i) Detection of “DNA ladders,” that is, multiples of 185 bp	Gel electrophoresis	Semiquantitative
	(ii) TUNEL	FM, FACS	Semi-quantitative
	(iii) COMET	Single cell gel electrophoresis	Semi-quantitative
	(iv) SubG_1_ (cell cycle analysis)	FACS	Quantitative

Nuclear fragmentation	For example, DAPI, Hoechst-33342 DNA-stained nuclei	FM	Quantitative

Membrane blebbing	Morphological changes	Phase contrast LM	Semi-quantitative

Caspase activation	Fluorometric/luminometric detection of cleavage of artificial caspase substrates	Microplate reader, FM, FACS	Quantitative, single-cell analysis via FACS

PSer exposure	Antibody staining	FM, FACS	Quantitative, single-cell analysis via FACS

Mitochondrial cyt-c release	Subcellular fractionation and immunodetection	Western blotting	Semi-quantitative

Mitochondrial ΔΨ breakdown	Fluorochrome-based assessment of mitochondrial ΔΨ	FM, FACS	Semi-quantitative (within cells), quantitative for comparison between cell populations

Membrane integrity, release of intracellular material^c^	(i) Detection of necrosis-associated plasma membrane breakdown via PI staining	FM, FACS	Quantitative, single-cell analysis via FACS
	(ii) Biochemical assay for LDH enzyme release from necrotic cells	Microplate reader	Quantitative

Cyt-c: cytochrome c; DAPI: 4′,6-diamidino-2-phenylindole; ΔΨ: mitochondrial membrane potential; FACS: fluorescence-activated cell sorter; FM: fluorescence microscopy; LM: light microscopy; PI: propidium iodide; PSer: phosphatidylserine; TUNEL: terminal deoxynucleotidyl transferase-mediated dUTP nick end labeling.

^a^Selection of methods is focused on *in vitro* experimentation (cell culture).

^b^Based on the author's experience.

^c^These methods address specific necrosis-associated cellular changes.

## Abbreviations

O_2_^∙−^: Superoxide anion
ABS: Absorbance
ATP: Adenosine 5′-triphosphate
BrdU: 5-bromo-2′-deoxyuridine
Caspase: Cysteine-dependentaspartate-specificprotease
CFU: Colony-forming unit
cyt-c: Cytochrome c
DAPI: 4′,6-diamidino-2-phenylindole
DC: Dark control
∆Ψ: Mitochondrial membrane potential
DMAX: 9-[2-(3-carboxy-9,10-dimethyl) anthryl]-6-hydroxy-3H-xanthen-3-one
DMPD: N,N-dimethyl-p-phenylenediamine
DMSO: Dimethyl sulphoxide
DPA: 9,10-diphenylanthracene
DPBF: 1,3-diphenylisobenzofuran
EDTA: Ethylenediaminetetraacetic acid
ET: Electron transfer
FACS: Fluorescence-activated cell sorting
FB/CS: Fetal calf/bovine serum
FI: Fluorescence intensity
FM: Fluorescence microscopy
HO^*∙*^: Hydroxyl radicals
LM: Light microscopy
LOC: Light-only control
MRSA: Methicillin-resistant *Staphylococcus aureus *
MTT: 3-(4,5-Dimethylthiazol-2-yl)-2,5-diphenyltetrazolium bromide
ORAC: Oxygen-radical absorbance capacity
PARP: Poly(ADP-ribose) polymerase
PDI(B): Photodynamic inactivation (of bacteria)
PDT: Photodynamic therapy
PeT: Photo-induced electron transfer
PI: Propidium iodide
PS: Photosensitizer
PSer: Phosphatidylserine
ROS: Reactive oxygen species
SOSG: Singlet Oxygen Sensor Green
TMRE: Tetramethylrhodamine ethyl ester
TMRM: Tetramethylrhodamine methyl ester
TRAP: Radical-trapping antioxidant parameter method
TUNEL: Terminal deoxynucleotidyl transferase-mediated dUTP nick end labeling
UTC: Untreated control
WST: Water-soluble tetrazolium salt
XTT: 2,3-bis-(2-methoxy-4-nitro-5-sulfophenyl)-2H-tetrazolium-5-carboxanilide.
